# Synthesis of Zeolite from Carbothermal Reduction Electrolytic Manganese Residue for the Removal of Macrolide Antibiotics from Aqueous Solution

**DOI:** 10.3390/ma11112133

**Published:** 2018-10-30

**Authors:** Xuli Li, Yue Zeng, Fangyuan Chen, Teng Wang, Yixin Li, Yuchi Chen, Haobo Hou, Min Zhou

**Affiliations:** 1 School of Resource and Environment Science, Wuhan University, Wuhan 430072, China; 2015102050038@whu.edu.cn (X.L.); 2017202050097@whu.edu.cn (Y.Z.); Faye.C@whu.edu.cn (F.C.); 2012301620006@whu.edu.cn (Y.L.); cycglacier@163.com (Y.C.); 2 Hubei Environmental Remediation Material Engineering Technology Research Center, Wuhan University, Wuhan 430074, China; 3 School of Environmental Engineering, Wuhan Textile University, Wuhan 430200, China; 2018001@wtu.edu.cn

**Keywords:** adsorption, electrolytic manganese residue, macrolide antibiotics, zeolite

## Abstract

Zeolite analcime (EMANA) was synthesized through the hydrothermal method by using carbothermal reduction electrolytic manganese residue (CR-EMR). The structural properties of EMANA and CR-EMR were studied using various characterization techniques. After hydrothermal synthesis, the CR-EMR became super-microporous, and the surface area increased by 4.76 times than before. Among the various synthesized zeolites, 6 h-synthesized EMANA was selected as the best adsorbent for macrolide antibiotics in aqueous solution. The adsorption performance of EMANA on the adsorption capacity was examined by using various experimental parameters, such as contact time (0–24 h), initial concentration (50–300 mg/L), temperature (30–50 °C) and pH (3–13). The experimental results were also analyzed by the Langmuir and Freundlich adsorption models, with the latter obtaining better representation. The adsorption process could be described well by the pseudo-second-order model, even under a low concentration (50 mg/L). This result suggests that the adsorption process of macrolide antibiotics is due to chemisorption. According to the Fourier Transform infrared spectroscopy (FT-IR) results, the adsorption of zeolite was mainly due to its hydroxyl group, which played an important role during the adsorption process. Moreover, EMANA is more suitable for treatment of roxithromycin (ROX) than azithromycin (AZM), because ROX has more adsorption sites for the hydroxyl group.

## 1. Introduction

Manganese is an essential element in steel and aluminum alloys. Manganese has become an irreplaceable element for the development of electrochemical energy storage devices, as new energy materials replace fossil fuels. Mn-containing steel and aluminum alloy is widely used in railway tracks, safes, beverage cans, and kitchenware [1]. Electrolytic manganese dioxide (EMD) is one of the most widely used cathode materials for alkaline batteries, lithium manganese primary batteries, and super capacitors [2]. To date, the growth in the EMD market is robust due to the increase in global demand for multiple battery requirements in areas of mobile communications, cameras and watches. The continuous researches on the development of less expensive battery cathode materials indicate that LiNi_x_Co_y_Mn_z_O_2_ (NCM) has similar or higher achievable specific capacity than lithium cobalt oxide and similar operating voltage but has low cost because of the reduced Co content [3]. LiNi_0.33_Co_0.33_Mn_0.33_O_2_ is widely used in the battery market, due to its capacity over 200 mAh/g [4]. Additional demands for EMD are also likely to come from the use of EMD in hybrid electric vehicles (HEVs). As the environmental impact of traditional cars continues to cause problems in densely populated cities, the increased usage of HEVs to minimize pollution levels seems inevitable [5].

In 2016, the electrolytic metal manganese output in China was 1.15 million tons, accounting for over 98.5% of the global supply [6]. For every metric ton of its product, the electrolytic metal manganese industry discharges 8–10 metric tons of residue [7]. All these findings defined the electrolytic metal manganese industry as an industry with a high level of resource consumption and large quantities of waste discharge. This situation made a significant variety of pollutants into the surrounding environment. High concentrations of manganese in residue constitute huge potential environmental risks [8]. The increased environmental and resource costs have resulted in closure of electrolytic metal manganese production in France, Japan and the USA, leaving only two countries in the world (China and South Africa) to produce electrolytic metal manganese from 2002 onwards. With the increasing demand for manganese, low-grade manganese ore has also been added to the production process. The electrolytic manganese companies in China exhibit three distinguishing features: low-grade manganese ores used as raw material (less than 40%), hazardous additives (selenium and chromium) used in the process, and backward automation level [9]. The main raw manganese ores are rhodochrosite and pyrolusite, which are ubiquitous, with grade below 40% on average [10]. Two different raw materials are produced with two different kinds of slag. The electrolytic manganese residue (EMR) was produced from leaching rhodochrosite ore with sulfuric acid before electrolytic process. The carbothermal reduction electrolytic manganese residue (CR-EMR) was produced from pyrolusite ore by reduction roasting with coal followed by leaching roasted slag with sulfuric acid. Both methods provide qualified solution containing manganese sulfate for further electrowinning process. The production process of electrolytic manganese is clear, but that of EMR and CR-EMR has not gained enough attention. Given the lack of mature resource recovery technology, these slags are generally stockpiled in residue galleries without pretreatment, and rainfall causes the escape of leachate from these holding ponds [11]. Previous studies mainly focused on the recovery and use of heavy metals in EMR, but neglected the large proportion of silicon and aluminum elements. These studies used electrochemical methods [12], acid leaching [13], and bioleaching [8] to extract manganese from EMR. EMR can also be used for brick-making materials [10], fillers in sulfur concrete [14], and cementitious materials [15]. However, building materials are not in a shortage. The building materials produced from waste residue are not very popular in the market, because the quality is not as good as the products being sold. Several researchers have paid attention to the large amount of silicon in EMR and activated silicon in EMR by mechanical grinding-roasting for plants to absorb [16]. However, the CR-EMR already went through a calcination temperature of 800–1200 °C, and the silicon had crystallized and became inactive. These factors made it more difficult than EMR to be used. Few studies have explored the idea of turning CR-EMR into effective materials. Overall it is extremely urgent to find a way to deal with CR-EMR.

Pharmaceuticals in aquatic environments are contaminants of emerging concern, not only due to their potential for ecotoxicological risks but also because of their continuous emissions to the environment [17]. Among the various pharmaceuticals, macrolide antibiotics hold special importance because of their extensive use in human therapy, and veterinary medicine, as well as in promoting the growth of animals in livestock production [18]. The first watch list of 10 substances/groups of substances refers to three macrolide antibiotics (erythromycin, azithromycin (AZM) and clarithromycin) was amended in the Decision 2015/495/EU of 20 March 2015 for European Union-wide monitoring [19]. Given the large Chinese population with their high consumptions, a nationwide survey indicated that macrolide antibiotics were the most frequently detected in 14 municipal wastewater treatment plants distributed across China [20]. Roxithromycin (ROX) and AZM were the most abundant macrolide antibiotics in the sample of influent, effluent, and excess sludge. Macrolide antibiotics cannot be completely removed by wastewater treatment plants; thus, they can persist in the environment at least for a year, or even for various years and become biologically active through accumulation [21]. This causes a potential risk for aquatic and soil organisms due to the presence of trace concentrations of these bioactive compounds [22]. Therefore, it is important to find a way to eliminate macrolide antibiotics. In the previous study, ROX was removed mainly by adsorption without causing biodegradation [23]. This suggests that adsorption can be widely applied for the removal of organic compounds. Although activated carbon is the most commonly used sorbent, it still has some limitations in application, such as flammability, difficulty of regenerating adsorbed high-boiling-point organics and high cost [24]. Unlike activated carbon, zeolite is a hydrated, crystalline, and microporous aluminosilicate structured into three-dimensional networks of TO_4_ (T = Si, Al) tetrahedra joined at the corners by oxygen atoms, which indicates that it has a significant number of intermolecular channels and cavities and rich ion-exchange chemistry ability [25]. Zeolite can be synthesized using leaching residue as Si and Al sources [26]. By using CR-EMR as a raw material, the cost of zeolite can be reduced, making it an effective alternative adsorbent. 

This study recycled CR-EMR into low-cost adsorbent (zeolite analcime) for the removal of macrolide antibiotics from aqueous solution. Two kinds of macrolide antibiotics, ROX and AZM, were selected as contaminants to represent the adsorption capacity of analcime. The adsorption capacity of zeolite was evaluated by using the experimental results of initial concentration, contact time, pH value, and electric potential of the pollutant solution. The adsorption mechanism is described by fitting models of adsorption isotherm and kinetic data. Fourier Transform infrared spectroscopy (FT-IR) was used for functional group analysis. In our study, we reduced the amount of typical wastes generated by the lack of manganese resources, thereby reducing the cost of the residue and the ecotoxicological risks of pharmaceuticals in the aquatic environment.

## 2. Materials and Methods

### 2.1. Materials

The CR-EMR sample was obtained from CITIC Dameng Mining Industries Reagent Co., Ltd., Nanning, Guangxi Province, China and was used as raw material to synthesize zeolite in this study. Its chemical composition is shown in Table 1. Azithromycin (BR, 98% purity) was obtained from Shanghai Macklin Biochemical Co., Ltd., Shanghai, China. Roxithromycin (BR, ≥98% purity) was obtained from Aladdin Industrial Corporation, Shanghai, China. Ethanol absolute (AR, ≥99.7% purity) was obtained from Fangzheng Reagent Factory, Beichen District, Tianjin, China. Sodium hydroxide (AR, ≥96.0% purity), ethanedioic acid dehydrate (AR, 98% purity), sodium aluminate (CP, ≥41.0 content in Al_2_O_3_), and sulfuric acid (AR, 95.0–98.0% purity) were supplied by Sinopharm Chemical Reagent Co., Ltd., Shanghai, China, and used as received.

### 2.2. Synthesis of Zeolite from CR-EMR

Before the synthesis of zeolite, CR-EMR was dried in a drying oven and then ball-milled. First, 10.0 g of the residue was reacted with 15 mL 0.8 mol·L^−1^ hydrogen peroxide solution at 90 °C for 2 h, and then 5.7 g of oxalic acid was added into the solution. Here, oxalic acid is used as a chelating agent to turn metals into trioxalatoferriates which stays in the solution. The slag and filtrate were seprated by filtration. The filtrate contained valuable metals such as Fe and Mn, and kept for further use. A mixture of 4.8 g Na_2_CO_3_ and 6 g of the leaching slag was milled and fused in a corundum crucible at 750 °C for 2 h. The resultant fused mixture was cooled and milled again. 

Based on a previous study [27], zeolite was synthesized using the fused mixture, and the chemical composition is listed in Table 1. Zeolite was prepared by hydrothermal synthesis. First, 10.0 g of fusion mixture was dissolved in 40 mL distilled water, and then 0.48 g sodium hydroxide powder and 0.2 g sodium aluminate powder were added into the solution followed by stirring and aging for 2 h at room temperature until being homogenized. The slurry was transferred into a Teflon-lined autoclave and heated at 180 °C for various time durations. Finally, the precipitates were filtered, washed repeatedly with distilled water, and dried at 105 °C.

The elemental composition of CR-EMR and synthesized zeolite was determined by standardless quantitative analysis method using X-ray fluorescence spectrometer (XRF, model S4 Pioneer, Bruker AXS, Karlsruhe, Germany). The mineralogical analysis was done using X-ray diffraction (XRD, model D8 Advance, Bruker AXS, Karlsruhe, Germany). The surface morphology of synthesized zeolite was studied by field emission scanning electron microscope (FE-SEM, model SIGMA, Carl Zeiss, Oberkochen, Germany) and scanning electron microscope (SEM-EDS, model Quanta 200, FEI, Eindhoven, Netherlands). Total surface area, pore volume, and pore diameter of CR-EMR and synthesized zeolites were determined by conducting nitrogen adsorption/desorption experiment at −195.85 °C using multi-station automatic specific surface and porosity tester (BET, model Belsorp-miniII, MicrotracBEL Corp., Osaka, Japan). The zeta potential was done by a high-performance two-angle particle size and molecular size analyzer (model Zetasizer Nano ZSE, Malvern Panalytical, Malvern, UK). Fourier transform IR spectrometry (FT-IR, model Nicolet 5700, Thermo Electron Scientific Instruments Corp., Waltham, MA, USA) was used to identify the functional groups inherited by synthesized zeolite.

### 2.3. Adsorption Studies

In total, 50 mL macrolide antibiotic solutions with varying initial concentrations (50–100 mg/L) was placed in a centrifuge tube, wherein 0.1 g of the prepared zeolite was added and continuously agitated at 200 rpm in a water bath shaker at room temperature to perform batch adsorption experiments. A portion of the solution was collected at predetermined time intervals for kinetics and at equilibrium time for isotherms. After a given time, the contents of the beakers were separated using a centrifuge (5000 rpm). Initial and final concentrations of macrolide antibiotics were obtained by adding H_2_SO_4_, and then absorbance measurements were performed at 481 nm by using UV/visible double beam spectrophotometer (Model UV 1700, Sjimadzu, Kyoto, Japan). 

The equilibrium adsorption capacity *q_e_* (mg/g) was calculated using the following equation.

(1) $$q_{e}=\frac{\left(C_{0}-C_{e}\right)V}{W}$$

where *q_e_* is the amount of macrolide antibiotics adsorbed per unit mass of adsorbent (mg/g), *C*_0_ is the initial macrolide antibiotics concentration (mg/L), *C_e_* is the macrolide antibiotic concentration (mg/L) at equilibrium, *W* is the mass of adsorbent on a dry basis (g), and *V* is the solution volume (L).

To determine the effect of pH on macrolide antibiotic adsorption, 50 mL of macrolide antibiotic solutions with pH ranging from 3 to 13 (adjusted using 0.1 mol/L NaOH and H_2_SO_4_) each having initial concentrations of 100 mg/L were placed in Erlenmeyer flasks, to which 0.1 g of the prepared zeolite was added.

The adsorption kinetics for adsorbents have been previously described by pseudo-first-order and pseudo-second-order reactions [28] and intraparticle diffusion model [29]. The linearized integral forms of these models are as follows:

(2) $$ln\left(q_{e}-q_{t}\right)=lnq_{e}-k_{1}t$$

(3) $$\frac{t}{q_{t}}=\frac{1}{k_{2}q_{e}^{2}}+\frac{t}{q_{e}}$$

(4) $$q_{t}=k_{i}t^{0.5}+C$$

where *k*_1_ (min^−1^) and *k*_2_ (mg^−1^·min^−1^) are the pseudo-first-order and pseudo-second-order rate constants, respectively; *k_i_* (mg/g·h^0.5^) is the intraparticle diffusion rate constant; and *q_e_* and *q_t_* (mg/g) are the amounts of macrolide antibiotics adsorbed at equilibrium and at time (*t*), respectively.

## 3. Results and Discussion

### 3.1. Characteristics of the Zeolite EMANA

The chemical compositions of materials and products are shown in Table 1. From the result of CR-EMR, many metals, such as Fe and Mn, were found. To make the best use of CR-EMR, we need to retrieve rare metals. As for Fe, we could neither find any crystal structure of ferri salt nor determine its valence state. Reportedly, during zeolite crystallization, both crystallinity and crystal size decrease with the increase of iron content [30]. The free Fe species influence the normal crystallization of zeolite and induce the formation of the impurities, such as ferric oxide, and amorphous silicon oxide at low ratio of SiO_2_/Fe_2_O_3_ [31]. We chose hydrogen peroxide to make sure all iron turn into Fe (III) and then used oxalic acid as a reducing agent to turn iron into trioxalatoferrate, which stays in the solution.

**Figure 1:**
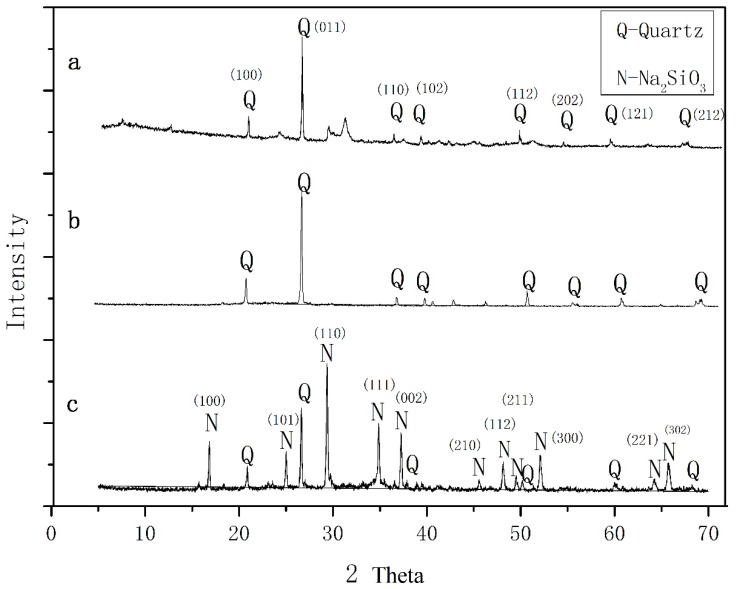
XRD patterns of (**a**) CR-EMR, (**b**) acid washed residue, (**c**) fused mixture.

The XRD patterns of the zeolite synthesized materials are shown in Figure 1. As can be seen from Figure 1a, most SiO_2_ in CR-EMR were in the form of quartz. As the materials of zeolite synthesized, we need activated silica, not the crystal type of quartz in CR-EMR. We made a mixture of Na_2_CO_3_ and milled and fused the leaching slag. The diffraction intensities of quartz showed an evident decrease, indicating that quartz turned into sodium silicate, which contributed to the synthesis of zeolite. Zeolitic materials were not detected in the XRD pattern of the fused mixture.

**Figure 2:**
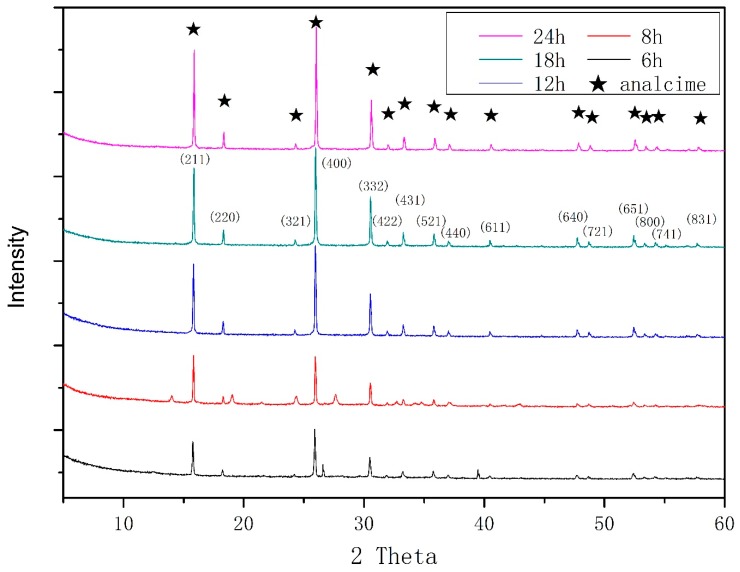
XRD patterns of the zeolite products obtained after varying the crystallization time.

**Figure 3:**
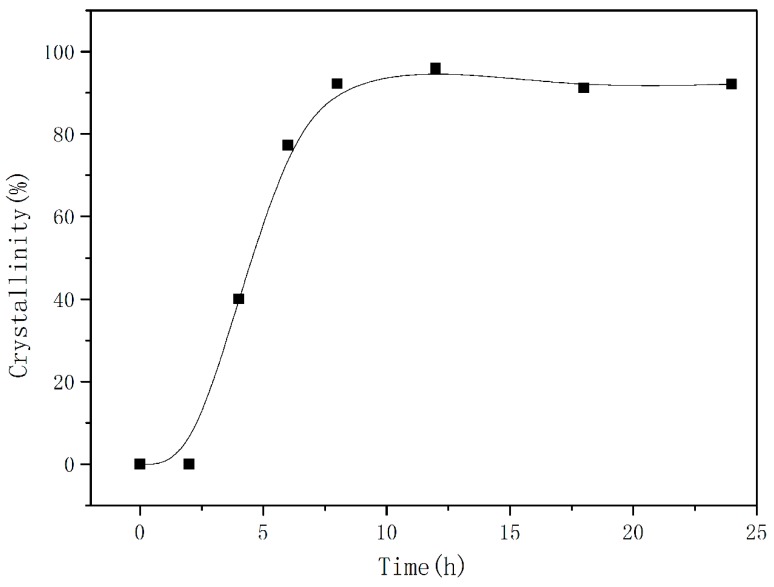
The crystallinity curve of synthesized zeolite EMANA.

To investigate the effect of time on zeolite crystallization, we maintained the basic conditions and only changed the crystallized time (Figure 2). At a crystallization time of 6 h, the major crystalline phase of the synthesized zeolite was identified as zeolite analcime (EMANA), with small amounts of wairakite and zeolite P. As time passed, a pure phase of zeolite EMANA was obtained. MDI Jade v6.0 (Material Data Inc., Livermore, CA, USA) was used to calculate the degree of crystallization (Figure 3). As seen from the figure, the curve has an “S” shape, which characterizes the typical growth curve of zeolite. This curve consists of the induction period, growth period, and equilibrium period. The crystallinity growth was slow in the induction period of 2 h synthesis, and increased rapidly from 2 h to 8 h. The equilibrium period of zeolite growth occurred after 8 h.

**Figure 4:**
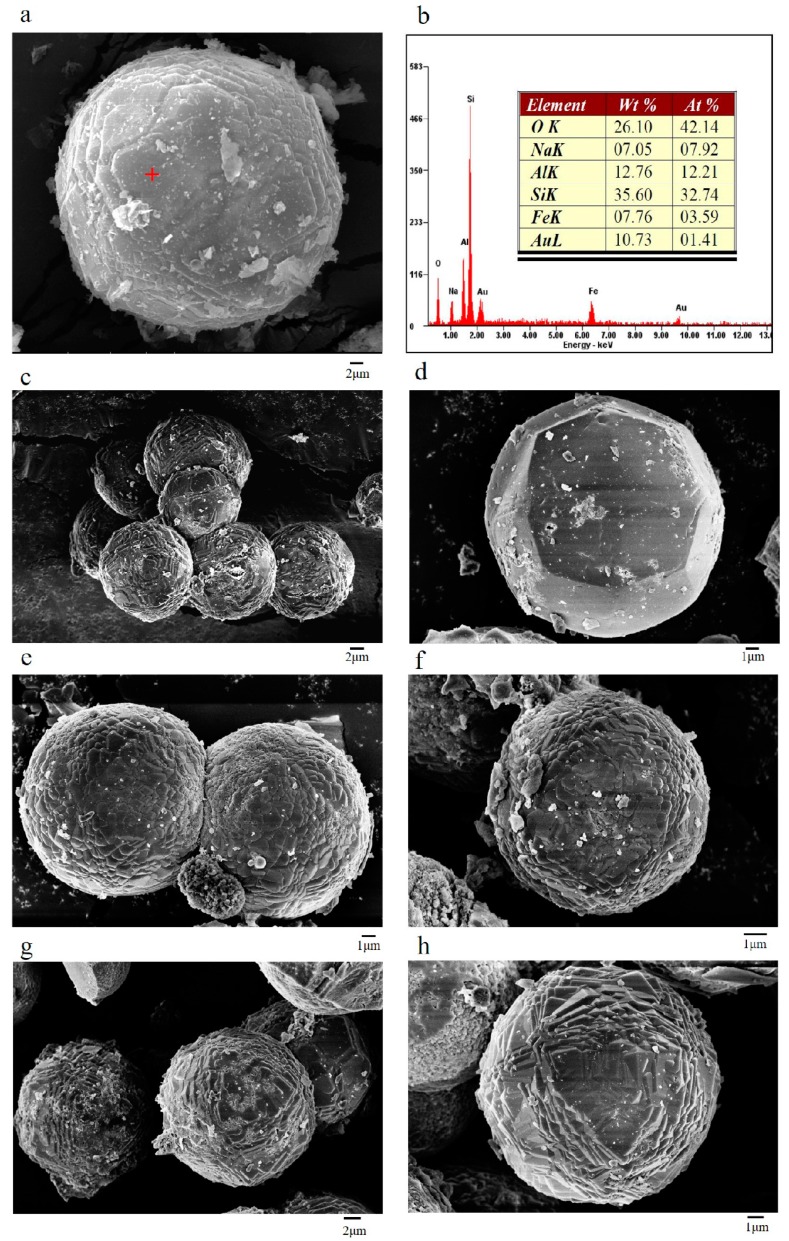
Scanning electron microscope (SEM-EDS) and field emission scanning electron microscope (FESEM) images of (**a**) 24 h synthesized zeolite EMANA, (**b**) energy dispersive spectrometer (EDS) spectrums of spot 1 in (**a**), (**c**) and (**d**) 6 h synthesized zeolite EMANA, (**e**) and (**f**) 8 h synthesized zeolite EMANA, (**g**) and (**h**) 12 h synthesized zeolite EMANA.

The scanning electron microscope (SEM-EDS) and field emission scanning electron microscope (FESEM) images of synthesized EMANA taken at different time points are shown in Figure 4. After 6 h of hydrothermal synthesis, the crystal was preliminarily formed. Figure 4c–h shows that with the extension of hydrothermal synthesis time, upon the formation of zeolite, the particles enlarged and the surface became rough, which indicates the deposition of clusters of zeolite crystals. Usually, the zeolite analcime (ANA) has an Si/Al ratio of 1.8–2.8. From the results of energy dispersive spectrometer (EDS), we obtained an Si/Al ratio of 2.68. From the result of X-ray fluorescence (XRF), the Si/Al ratio was 3.52. The reason for the difference in the two Si/Al ratios is because the XRF result is the atomic ratio of the total silicon to the total aluminum and the EDS represents the ratio of Si/Al on the zeolite crystal skeleton.

**Figure 5:**
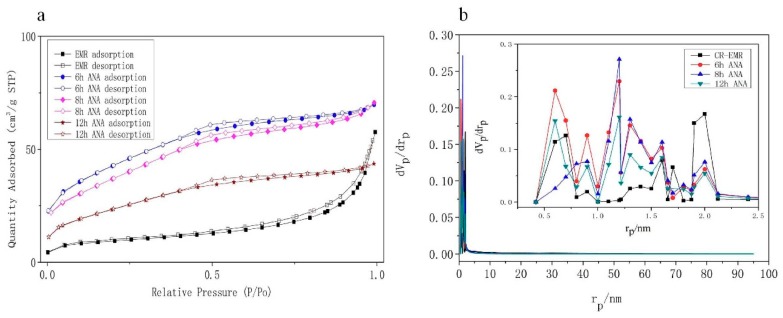
(**a**) N_2_ adsorption-desorption isotherm and (**b**) pore size distribution of CR-EMR, 6 h synthesized zeolite EMANA, 8 h synthesized zeolite EMANA, and 12 h synthesized zeolite EMANA.

N_2_ adsorption was carried out for the synthesized EMANA sample to evaluate the micropollutant uptake capacity. In Figure 5, zeolite EMANA exhibits reversible type I-B isotherm, which is one of the main characteristics of micro-mesoporous complex materials. The pore diameter distribution of this micro-mesoporous complex material is wide and may have narrow mesoporous structure at the same time. From Figure 5a, the slight adsorption–desorption hysteresis is for 0.35 < *P*/*P*_0_ < 0.95, type H4-shaped hysteresis loop, which is a typical curve of solid with narrow fissure hole type. The surface area of zeolite EMANA was calculated using the Brunauer-Emmett-Teller model listed in Table 2. In Figure 5b, the pore size distribution of the synthesized zeolite EMANA was calculated using the MP desorption model, showing that the effective pore diameter is concentrated at 0.5–2.0 nm, which is very different to the mean pore diameter. This result is possibly due to the accumulation of disordered crystal, which can also be seen in Figure 4. Originally, the pore diameter distribution of CR-EMR was concentrated at approximately 2 nm, and the surface area was 29.049 m^2^/g. After hydrothermal synthesis, the micropore size increased, mostly concentrated at 0.5–1.5 nm, and the surface area increased to 138.4 m^2^/g. These coarse micropores (0.5–2 m) were divided into independent types of micropores, called super-microporous [32]. When the filling degree of adsorption volume is low, the super-microporous adsorption can be regarded as the single or multilayer surface coverage of the adsorbent molecules.

### 3.2. Choice of Asorbent

Adsorption uptake of various synthesized zeolite EMANA was compared for ROX and AZM. On one hand, the crystallinity could reflect the purity of the zeolite and 8 h was the equilibrium duration of zeolite growth. Shortening the synthesis time can also reduce the cost of waste use. On the other hand, the large specific surface area is an advantage because several adsorbing contact sites are available for the attachment of macrolide antibiotics. The high specific surface area is one of the contributing factors to the adsorption of pollutants. Therefore, the 6 h-synthesized zeolite EMANA with the shortest synthesis time and the highest specific surface area among all the three composites was selected as the best adsorbent in the following studies.

### 3.3. Effects of Initial Concentrations and Contact Time on Adsorption

**Figure 6:**
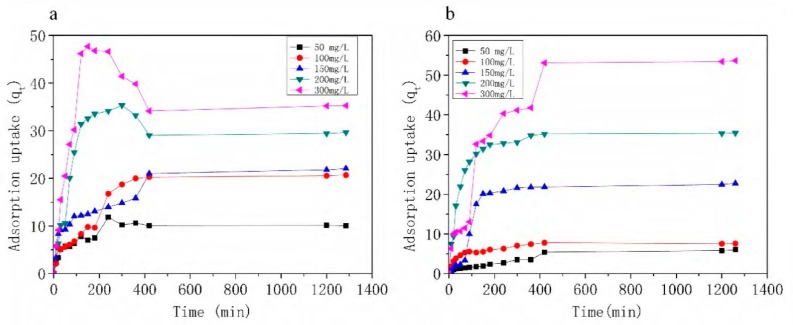
Effects of initial concentration and contact time on (**a**) azithromycin (AZM) and (**b**) roxithromycin (ROX) adsorption onto 6 h synthesized zeolite EMANA (V = 100 mL, W = 0.20 g, shaking speed of 140 rpm, and temperature = 30 °C).

Figure 6 shows the results of the adsorption uptake of macrolide antibiotics for 6 h-synthesized zeolite EMANA versus contact time at different initial concentrations (50, 100, 150, 200, and 300 mg/L). Macrolide antibiotics adsorption was initially fast but lightly decreased and finally became gradually slow as equilibrium approached 400 min. This result may be because of the saturation of the active sites at the early stages of adsorption. Many vacant active sites of 6 h-synthesized zeolite EMANA are accessible, but as time increases, the adsorbent surface adsorption point is occupied, thus slowing down the adsorption process [33]. For further experiments, more than 400 min was set to be sufficient for macrolide antibiotics to achieve uptake equilibrium. As the initial concentration was enhanced from 50 mg/L to 300 mg/L, the adsorbed amount of AZM increased from 10.13 mg/g to 35.27 mg/g, and the adsorbed amount of ROX increased from 6.03 mg/g to 53.65 mg/g. With this adsorbent dosage, the adsorbed amount may have reached the performance limitation of this material. If the adsorbent dosage is increased, the adsorption effect may be improved. This result indicated that the removal performance is affected by the initial concentration. Unlike ROX, even at high initial concentration of AZM, the adsorbent has certain desorption phenomenon. We speculated that this desorption phenomenon was caused by physical adsorption, which can be done quickly and reversibly. As from the result of adsorption isotherm analysis, the whole adsorption process was dominated by chemical adsorption. We can get support from the following results of FT-IR. The FT-IR result shows the hydroxyl group on the zeolite surface plays an important role during the adsorption process.

### 3.4. Effects of pH and Zeta Potential

**Figure 7:**
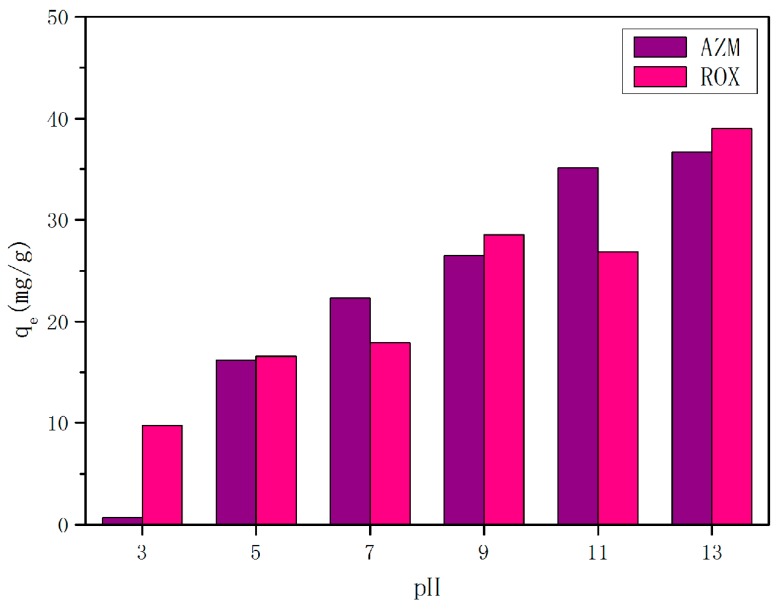
Solution pH versus macrolides antibiotics uptake of 6 h synthesized zeolite EMANA (V = 100 mL, W = 0.20 g, initial concentration = 200 mg/L, shaking speed = 140 rpm, and temperature = 30 °C).

**Figure 8:**
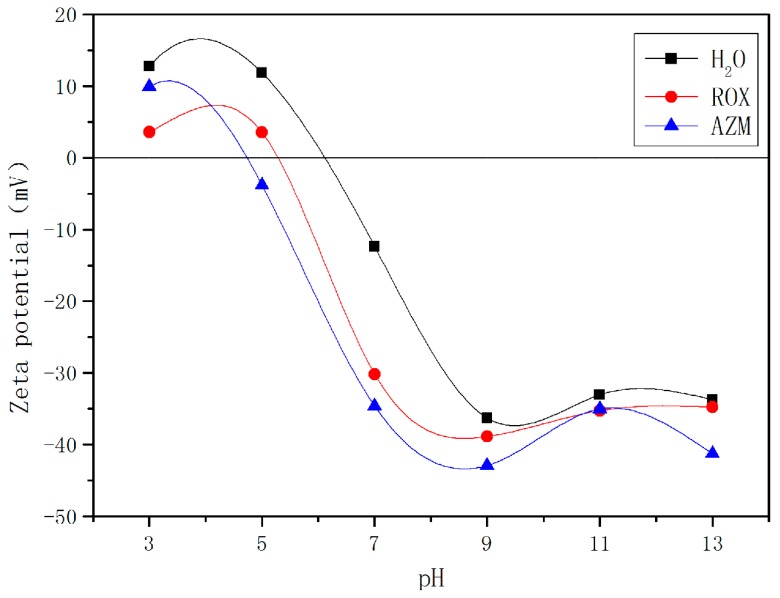
Zeta potential of 6 h synthesized zeolite EMANA before and after adsorption.

The adsorption is found to be pH dependent as pH affects the speciation of macrolide antibiotics and surface charge of the adsorbent [34]. Initial solution pH values ranging from 3 to 13 and its effect on macrolide antibiotic adsorption over 6 h-synthesized zeolite EMANA are presented in Figure 7. Clearly, the pH of the solution greatly influenced the adsorption process of macrolide antibiotics, and the uptake of AZM and ROX was increased from 0.67 mg/g to 36.69 mg/g and 16.59 mg/g to 49.04 mg/g, respectively, when pH increased from 3.0 to 13.0. This situation was consistent with the conclusions of a previous study [35]. As a result, the adsorption capacity of 6 h-synthesized zeolite EMANA is low at low pH values, specifically below 5, due to electrostatic repulsion between macrolide antibiotics and 6 h-synthesized zeolite EMANA. The electrostatic attraction between the drug and surface of adsorbent was also gradually increased to the highest amount of adsorption at pH greater than 9, which agreed well with the zeta potential result (Figure 8). The increased negative charge was attributed to the presence of the hydroxyl groups [36]. The boundary of particle dispersion stability in water phase is generally considered to be +30 mV or −30 mV. If all particles have zeta potential higher than +30 mV or lower than −30 mV, the dispersion system should be stable. As pH values increase, the zeta potential definitely becomes lower than −30 mV, which is beneficial for the adsorption process. In addition, with the decrease in pH, metal uptake decreased, which may be due to the partial destruction of zeolite crystal at low pH [37]. In consideration of the cost, the pH value of 9 was selected.

### 3.5. Adsorption Kinetics Analysis

**Figure 9:**
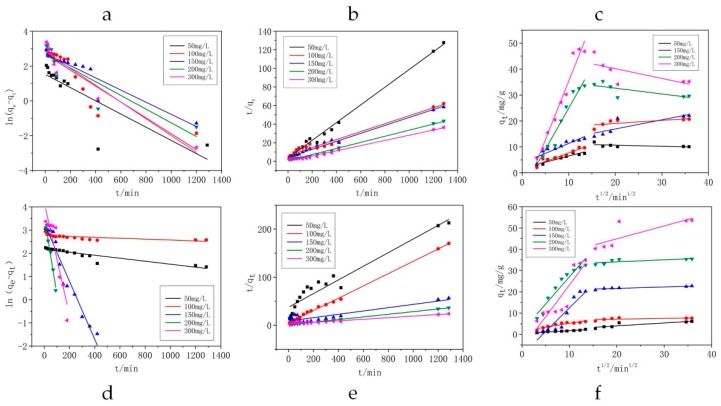
Modeling of (**a**,**b**,**c**) AZM and (**d**,**e**,**f**) ROX adsorption data via kinetic simulations: (**a**) and (**d**) pseudo-first-order, (**b**) and (**e**) pseudo-second-order, (**c**) and (**f**) intraparticle diffusion model.

To understand the adsorption process, it is important to study the adsorption kinetics. The adsorption rate constants (*k*_1_, *k*_2_, *k_i_*) and the corresponding correlation coefficients (R^2^) are shown in Table 3. The linear fitting of these three models is shown in Figure 9. As we can see from the result, the pseudo-second-order model fits the experimental data for all systems better than the other two models. The calculated value of *q_e_* (cal) by using the pseudo-second-order model matched closest with the *q_e_* (exp) value. The correlation coefficient for the pseudo-second-order kinetic model is greater than 0.9, indicating the applicability of this kinetic equation. Furthermore, under the condition of low concentration (50 mg/L), the pseudo-second-order model still had excellent fitting degree. This result suggested that the adsorption process of macrolide antibiotics is due to chemisorption, which is the assumption behind the pseudo-second-order model.

The steps of adsorption process were studied with the intraparticle diffusion model. Figure 9c,f show that the two steps occur during the adsorption process. The first step corresponded to film diffusion, and the diffusion of macrolide antibiotic molecules from the solution to the external surface of the synthesized zeolite EMANA. The second step can be ascribed to the intraparticle diffusion stage. None of the plot passed through the origin, indicating that the intraparticle diffusion was not the rate limiting step [33]. *k_i_*_1_ was larger than *k_i_*_2_ but still lower than 0.9, suggesting that film diffusion only acts minimally on the first step of the adsorption process, and is not the determinant of the entire process.

### 3.6. Adsorption Isotherm Analysis

In this study, Langmuir and Freundlich isotherm models were chosen to analyze the macrolide antibiotic adsorption equilibrium. The Langmuir isotherm is valid for monolayer adsorption onto a surface with a finite number of identical sites [38]. It is expressed using the following equation:

(5) $$q=\frac{q_{m}bC_{e}}{1+bC_{e}}$$

The linearized form of the Langmuir equation is:

(6) $$\frac{C_{e}}{q}=\frac{1}{q_{m}b}+\frac{C_{e}}{q_{m}}$$

where *q_m_* and *b* can be determined from the linear plot of *C_e_*/*q* versus *C_e_*.

The Freundlich isotherm is expressed as follows:

(7) $$q_{e}=K_{f}C_{e}^{1/n}$$

The linearized form of the Freundlich equation is:

(8) $$\ln Q_{e}=\ln K_{f}+\frac{l}{n}\ln C_{e}$$

where *K_f_* (mg/g) (L/mg)^1/*n*^ is the Freundlich constant and 1/*n* is the heterogeneity factor. Moreover, *Q_m_* was calculated from Freundlich isotherm by using the Halsey equation [39]:

(9) Qm=Kf(C0)1n

**Figure 10:**
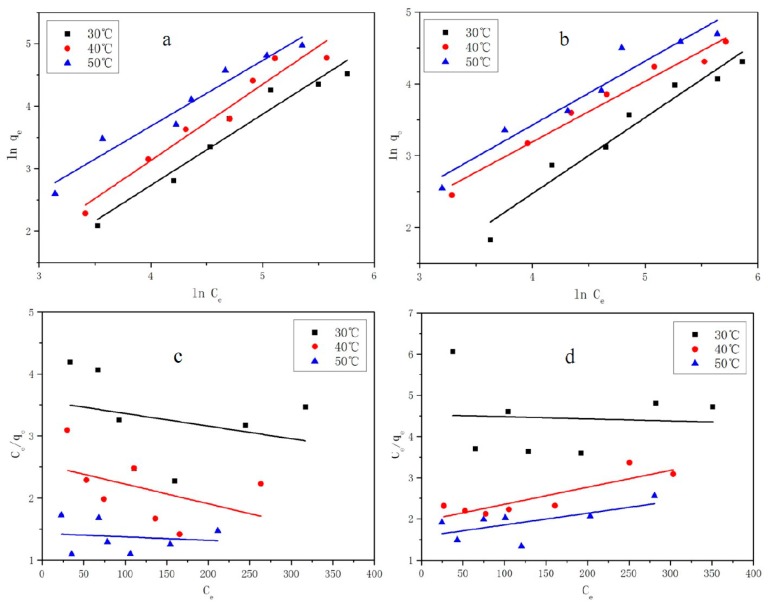
Langmuir and Freundlich adsorption isotherms for adsorption of (**a**,**c**) AZM and (**b**,**d**) ROX on EMANAN at different temperatures: (**c**) and (**d**) Langmuir,(**a**) and (**b**) Freundlich.

The values of the constants for the previous two models are shown in Table 4. From Figure 10, we can see the results confirming that the adsorption process fitted better with the Freundlich isotherm than the Langmuir isotherm because of the higher corresponding correlation coefficients. Similar results were reported for adsorption on analcime [34,40]. Physical adsorption can be either monolayer or multilayer, but the chemical adsorption is monomolecular, so it can be described by Langmuir model. These adsorption processes fit better with the Freundlich isotherm than the Langmuir isotherm, which also means during whole adsorption process is physical adsorption followed by chemical adsorption. The adsorption strength was related to the empirical parameter 1/*n* for the Freundlich isotherm adsorption model. In general, the adsorption process is easy to proceed when 0.1 < 1/*n* < 0.5. When 1/*n* > 2, it indicates that the adsorption reaction is difficult. In this experiment, 1/*n* is approximately 1, definitely lower than 2 under three temperature conditions, indicating that the synthesized zeolite EMANA is suitable for the removal of macrolide antibiotics. When the temperature increased the value of 1/*n* was reduced, which indicates that the increase in temperature will promote the adsorption reaction. Moreover, the Langmuir isothermal adsorption model mainly occurs when adsorbent concentration is low and is applicable to the fitting of homogeneous adsorption system, whereas the Freundlich model is applicable to the fitting of the heterogeneous adsorption system. Based on the Halsey equation, the maximum monolayer adsorption capacities of AZM and ROX for the synthesized zeolite ANA are 407.54 and 221.21 (mg/g) at 50 °C when *C*_0_ is 500 mg/L.

### 3.7. FT-IR Spectrometry Analysis

**Figure 11:**
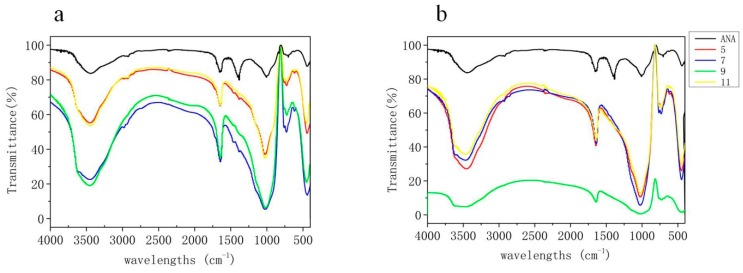
Fourier Transform infrared spectroscopy (FT-IR) spectrum of EMANA and after adsorption at different pH, (**a**) AZM (**b**) ROX.

The FT-IR spectrograms of the samples before and after the macrolide antibiotic adsorption are shown in Figure 11. We found the most common peak to zeolite in the synthesized zeolite EMANA at 443 and 1008 cm^−1^. These absorption bands are characteristic of the internal TO_4_ (T = Si or Al) vibrations, indicating the O–T–O asymmetrical stretching modes (1250–950 cm^−1^) and T–O bending modes (500–420 cm^−1^), respectively [41]. The band at 3447 cm^−1^ corresponds to hydroxyl stretching of water molecules present in zeolite and 1648 cm^−1^ is ascribed to hydroxyl bending and vibration of water entrapped in zeolite [42].

After ROX and AZM adsorption, the TO_4_ (T = Si or Al) vibrations at 1020 and 450 cm^−1^ still remain unchanged. This indicated that the structure of synthesized zeolite ANA stayed unchanged during the adsorption process, and the macrolide antibiotics had been adsorbed onto the surface of the synthesized zeolite ANA. The H–O–H deformation of water was observed at approximately 1640 cm^−1^ and water–water hydrogen bonding was observed at approximately 728 cm^−1^ [43]. This finding indicated that the hydroxyl group in the zeolite plays an important role during the adsorption process. From Figure 3c–e, the adsorption results of ROX were better than those of AZM. These outcomes can be explained by ROX containing two more methoxyl functional groups and ethoxylate than AZM, signifying that ROX has several adsorption sites for the hydroxyl group. Moreover, the peak of H–O–H deformation at 1648 cm^−1^ shifted to 1644 cm^−1^ after macrolide antibiotic adsorption, possibly attributed to the negatively charged surfaces of the adsorbent, wherein numerous –Si–O– existed and could provide adsorption sites for electrostatic interaction [33]. Furthermore, Chetan K. Chauhan demonstrated that the medium absorption band at 728 cm^−1^ indicates the wagging modes of vibration of the coordinated water and the metal–oxygen bond in the complex [44].

## 4. Conclusions

For the first time, the CR-EMR produced by reduction roasting with coal and leaching with sulfuric acid, was successfully recycled and used to make a low-cost adsorbent material. This process does not change the existing recycling process of electrolytic manganese production enterprises. While maximizing the benefits of recycling metals, this method can deal with the remaining waste residue. The zeolite EMANA with high crystallinity and surface area (from 29.05 m^2^/g to 138.4 m^2^/g) was successfully synthesized by hydrothermal method, as confirmed by XRD and SEM analyses. The EDS study confirmed that the ratio of Si/Al on the zeolite crystal skeleton is 2.68, which is characteristic to zeolite EMANA and these findings were supported by the XRD results. These characteristics verified EMANA to be the best candidates for application in adsorption of pharmaceuticals (macrolide antibiotics) in aquatic environments. The adsorption of macrolide antibiotics was found to be dependent on initial concentration, temperature, pH, and contact time. The adsorbed amount of macrolide antibiotics increased with increasing initial concentration, pH and temperature. The best representation of macrolide antibiotic adsorption data was obtained by the Freundlich model. According to the FT-IR results, the adsorption of zeolite is mainly due to the hydroxyl group in the zeolite, which plays an important role during the adsorption process. Moreover, EMANA is more suitable for dealing with ROX than AZM because ROX has two more methoxyl functional groups and ethoxylate than AZM, indicating that ROX contains several adsorption sites for the hydroxyl group.

## Figures and Tables

**Table 1:** Major chemical composition of carbothermal reduction electrolytic manganese residue (CR-EMR), acid washed residue, fused mixture and zeolite analcime (EMANA).

Sample	Chemical Composition (wt %)								
	SiO_2_	CO_2_	Fe_2_O_3_	SO_3_	Al_2_O_3_	MnO	K_2_O	CaO	Na_2_O
CR-EMR	38.75	15.80	14.94	10.80	8.12	6.40	1.74	1.32	0.10
Acid washed residue	59.97	27.15	2.51	3.33	3.68	1.04	0.9	0.16	0.05
Fused mixture	47.29	3.71	5.32	3.16	3.60	1.86	1.55	0.54	30.86
EMANA (24 h)	56.86	3.60	6.69	0.10	13.70	2.15	0.88	1.62	10.60

**Table 2:** The properties of particles of zeolite EMANA.

Material	BET Surface Area m^2^/g	Total Pore Volume cm^3^/g	Mean Pore Diameter nm
CR-EMR	29.05	0.08498	11.701
6 h synthesized zeolite ANA	138.4	0.1078	3.1143
8 h synthesized zeolite ANA	128.6	0.1089	3.3884
12 h synthesized zeolite ANA	81.43	0.06748	3.3149
24 h synthesized zeolite ANA	24.95	0.05687	9.1165

**Table 3:** Constants and correlation coefficients of pseudo-first order, pseudo-second order and intraparticle diffusion kinetic models for adsorption of macrolide antibiotics onto the EMANA.

Contaminant	Pseudo-First-Order Model						Pseudo-Second-Order Model				Intraparticle Diffusion Model (I)					Intraparticle Diffusion Model (II)			
	*C*_0_ mg/L	*q_e_* exp mg/g	*q_e_* cal mg/g	*k*_1_ × 10^4^ min^−1^	R^2^	SE	*q_e_* cal mg/g	*k*_2_ × 10^2^ mg/min	R^2^	SE	*C*_0_ mg/L	*k_i_*_1_ mg/(g·min^1/2^)	*C*_1_ mg/g	R^2^	SE	*k_i_*_2_ mg/(g·min^1/2^)	*C*_2_ mg/g	R^2^	SE
AZM	50	10.06	4.57	38.00	0.661	0.35	10.49	0.26	0.995	0.92	50	0.47528	1.71451	0.852	0.62	−0.04357	11.52809	0.149	0.80
	100	20.69	14.59	45.70	0.771	0.26	23.32	0.03	0.972	0.96	100	0.65600	1.09532	0.894	0.71	0.11943	16.64782	0.379	1.49
	150	22.05	15.18	35.00	0.878	0.14	23.31	0.04	0.986	0.67	150	0.77086	3.63343	0.796	1.21	0.35260	9.86107	0.654	2.74
	200	29.66	14.59	39.40	0.780	0.35	30.57	0.15	0.989	0.45	200	3.18475	−6.89820	0.942	2.50	−0.22403	37.13355	0.428	2.59
	300	35.26	16.95	48.20	0.920	0.24	35.84	0.80	0.990	0.37	300	4.51741	−9.81108	0.958	2.98	−0.37540	47.67356	0.368	4.78
ROX	50	6.03	8.77	6.42	0.850	0.04	7.04	0.05	0.927	0.52	50	0.13121	0.38335	0.94	0.10	0.13734	1.21127	0.149	0.97
	100	7.55	15.88	1.96	0.441	0.03	7.80	0.34	0.998	0.68	100	0.35932	1.57055	0.824	0.52	0.03410	6.44447	0.156	0.62
	150	22.71	26.41	1.25	0.950	0.17	28.99	0.01	0.816	0.21	150	2.21944	−9.77361	0.851	2.90	0.06936	20.19534	0.803	0.38
	200	35.47	36.27	3.40	0.981	0.11	36.42	0.09	0.999	0.10	200	2.55359	1.49440	0.913	2.48	0.09797	32.11436	0.449	1.10
	300	53.64	63.94	2.42	0.829	0.38	61.31	0.01	0.963	0.42	300	2.98133	−6.64054	0.777	4.97	0.60790	32.76858	0.575	5.49

**Table 4:** Langmuir and Freundlich isotherm parameters for the adsorption of macrolide antibiotics adsorption over 6 h EMANA.

Contaminant	Temperature (°C)	Langmuir Isotherm Model				Freundlich Isotherm Model			
		*q_m_* mg/g	*b* L/mg	R^2^	SE	*k_f_* (mg/g) (L/mg)^1/*n*^	1/*n*	R^2^	SE
AZM	30	−490.196	−0.0005	−0.102	0.53	0.162	1.138	0.937	0.58
	40	−314.465	−0.0013	0.049	0.39	0.176	1.218	0.939	0.58
	50	−1844.004	−0.0004	−0.176	0.20	0.590	1.052	0.945	0.45
ROX	30	−1921.74	−0.0001	−0.195	0.68	0.171	1.059	0.944	0.52
	40	242.13	0.0021	0.709	0.18	0.827	0.846	0.966	0.30
	50	352.11	0.0018	0.303	0.22	0.871	0.891	0.925	0.47
